# Acute myelocytic leukemia in a patient with hemophagocytic lymphohistiocytosis: A case report

**DOI:** 10.3892/ol.2014.2527

**Published:** 2014-09-12

**Authors:** DEFENG ZHAO, LIREN QIAN, JIANLIANG SHEN

**Affiliations:** Department of Hematology, Navy General Hospital, Beijing 100048, P.R. China

**Keywords:** hemophagocytic lymphohistiocytosis, leukemia, chemotherapy

## Abstract

Hemophagocytic lymphohistiocytosis (HLH), also known as hemophagocytic syndrome, is an aggressive hyperinflammatory condition characterized by prolonged fever, cytopenias and hepatosplenomegaly, as well as hemophagocytosis by activated, morphologically benign macrophages. HLH may be characterized into two forms, familial and secondary HLH. Familial HLH usually manifests in children with genetic abnormalities associated with the cytotoxic function of NK and T cells, whereas secondary HLH usually occurs in older patients in combination with an associated condition, such as infection or malignancy, without an identifiable genetic abnormality. Malignancy-associated hemophagocytic lymphohistiocytosis is mostly accompanied by lymphoid neoplasms. The present study reports a rare case of this syndrome in combination with acute myeloblastic leukemia (AML-M2), in a patient with clonal karyotypic abnormalities. The patient was successfully treated with chemotherapy comprising daunorubicin (40 mg/m^2^ i.v., days 1–3) and cytosine arabinoside (100 mg/m^2^, 1-h i.v. infusion, days 1–7). All clinical symptoms disappeared following chemotherapy.

## Introduction

Hemophagocytic lymphohistiocytosis (HLH), also termed hemophagocytic syndrome, is an aggressive hyperinflammatory condition characterized by prolonged fever, cytopenias and hepatosplenomegaly, as well as hemophagocytosis by activated, morphologically benign macrophages. There are two main types of HLH, familial HLH (FHLH) and secondary HLH. Familial HLH (FHLH) is an autosomal recessive syndrome with an estimated prevalence of 1/50,000 live births (1). Secondary HLH is a well-recognized entity and is associated with infections, autoimmune diseases, immune deficiencies, metabolic diseases, drugs or malignancies (2). The incidence of secondary HLH is unknown. Malignancy-associated hemophagocytic lymphohistiocytosis is mostly accompanied by lymphoid neoplasms. The present study describes a rare case of this syndrome in combination with acute myeloblastic leukemia (AML), in a patient with an abnormal karyotype, who was successfully treated with chemotherapy. Written informed consent was obtained from the the family of the patient.

## Case report

### Case presentation

**Figure 1:**
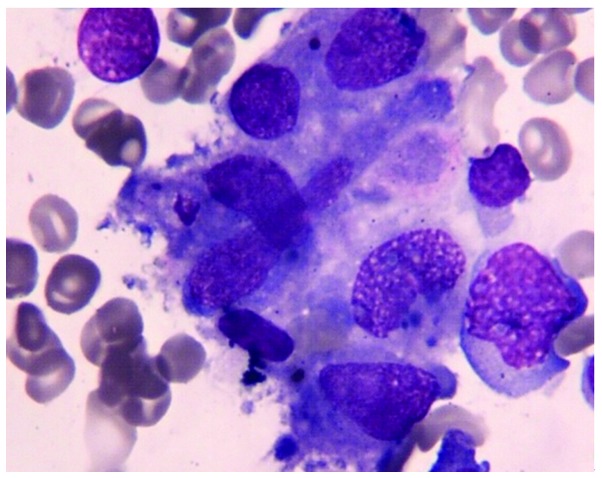
Bone marrow aspirate smear showed phagocytosis at the diagnosis of hemophagocytic lymphohistiocytosis (stain, hematoxylin and eosin; magnification, ×1,000).

A 61-year-old female presented to the Hematological Department of Navy General Hospital (Beijing, China) with a history of high fever for 30 days. The complete blood count showed pancytopenia; the white blood cell (WBC) count was 1.92×10^9^/l (normal range, 4–10×10^9^/l), the hemoglobin (Hgb) levels were 60 g/l (normal range, 120–150 g/l)and the platelet (PLT) count was 33×10^9^/l (normal range, 100–300×10^9^/l). Elevated levels of serum ferritin (11,966.7 μg/l; normal range, 11–360 μg/l) were detected. A reduced level of fibrinogen (Fg; 0.87 g/l; normal range, 1.5–4.0 g/l) and increased levels of lactate dehydrogenase (LDH; 1,617 U/l; normal range, 230–460 U/l), triglycerides (2.28 mmol/l; normal range, 1.7–2.25 mmol/l) and D-Dimer (6,779 μg/l; normal range, 0–300 μg/l) were detected. Serum antibody to Epstein-Barr virus (EBV) were negative, while serum antibodies to human immunodeficiency virus (HIV), hepatitis A, B and C (HAV, HBV and HCV), tubercle bacillus and hemococcidium were negative. Repeated blood cultures were negative. Bone marrow (BM) aspirate showed increased histiocytes (4.5%) with hemophagocytosis, and dysplasia in granulocytic and erythroid lineage. BM examination revealed a hypercellular marrow with 29% blasts accompanied by histiocytes with hemophagocytosis, but the erythroid progenitors were only 0.5% of total nucleated cells (Fig. 1). Morphology and flow cytometry studies showed no evidence of hematological malignancy. The levels of serum soluble interleukin 2 (IL-2) receptor (sCD25) in plasma of the BM were 44,000 pg/ml (normal levels, <6,400 pg/ml), while natural killer (NK) cell activity was 6.72% (normal range, 31.54–41.58%). G-banding analysis showed that these blasts had a chromosomal abnormality with 48,X, add(X)(p11),+3,der(7)t(1;7)(q11;p22),inv(12)(q15q24),add(14)(q32),t(14;19)(q32;q13),+18,add(21)(q22. CT scan examination revealed splenomegaly.

### Diagnosis

On the basis of both clinical and laboratory findings (fever, splenomegaly, cytopenias, hypofibrinogenemia, hemophagocytosis in the BM, hyperferritinemia, raised serum sCD25 levels and decreased NK-cell activity), a diagnosis of hemophagocytic lymphohistiocytosis HLH was therefore established. A bone marrow biopsy revealed a hypocellular marrow, which was composed of 25% myeloblasts, and thus the patient was simultaneously diagnosed with AML-M2.

### Treatment

The patient received chemotherapy for AML, comprising daunorubicin (40 mg/m^2^ i.v., days 1–3) and cytosine arabinoside (100 mg/m^2^, 1-h intravenous infusion, days 1–7). However, the treatment for HLH, according to the HLH 2004 protocol (6), was not started. Following the initial cycle of chemotherapy, clinical symptoms subsided. BM examination showed resolution of hemophagocytosis, although significant dyserythropoiesis was noted in the BM smears. The WBC and PLT counts, as well as the LDH and Fg levels, normalized. Hgb levels were also raised, but did not recover to normal levels. The serum ferritin levels declined gradually, but remained elevated (758 μg/l). The hemophagocytic syndrome was ameliorated after the first cycle of chemotherapy.

The patient achieved BM remission without hemophagocytosis after the second cycle of chemotherapy. Following consolidation therapy, comprised of mitoxantrone (4 mg/m^2^ i.v., days 1–3) and cytosine arabinoside (100 mg/m^2^, 1-h infusion, days 1–7), the patient developed pneumonia with fever. The patient succumbed to septic shock 4 months following the initial diagnosis of AML.

## Discussion

HLH has been traditionally classified as either primary familial HLH, with a genetic etiology, or secondary HLH, which is associated with malignancies, autoimmune diseases and infections (1). HLH is characterized by uncontrolled cytokine production secondary to underlying defective NK cell activity, resulting in persistent cytotoxic T-cell activation, macrophage proliferation and hemophagocytosis (1). Studies of cytokine levels in the blood and tissues of HLH patients have indicated persistently elevated levels of multiple pro-inflammatory cytokines during symptomatic disease, including IL-1β, tumor necrosis factor-α, IL-6, IL-8 and interferon gamma (1,4,5). It is currently considered that hypercytokinemia and hyperchemokinemia underlie the potentially fatal organ dysfunction in patients with secondary HLH. Elevated levels of sCD25, a marker of T-cell activity, have been shown to be correlated with the prognosis of HLH in children (6). In accordance with the International Histiocyte Society guidelines (7), five of the following eight diagnostic criteria are required for a diagnosis of secondary HLH: Fever, cytopenia of two cell lines, hypertriglyceridemia and/or hypofibrinogenemia, hyperferritinemia (>500 g/l), hemophagocytosis, elevated sCD25 levels, decreased NK cell activity, and splenomegaly. All criteria, with the exception of hemophagocytosis, were present in our patient.

Although a diagnosis of HLH was made in the present case, the cause remained unclear. Secondary HLH is frequently associated with an infectious etiology, including EBV, cytomegalovirus, HAV, HCV, HBV, herpes simplex virus, HIV, *Escherichia coli*, histoplasma and pneumocystis (8). The wide range of triggers of HLH has prompted researchers to stress the importance of identifying the underlying cause to enable targeted therapy (9). However, the present patient was extensively investigated for viral, bacterial and fungal infections, both in peripheral blood and BM samples, and was not found to have any of the described infections. HLH has been described in association with various types of hematological malignancies, particularly T-cell lymphoma, although there were proven or suspected infectious triggers in both cases (10,11). There have been two reported cases of AML and secondary HLH, one of which was associated with infection, another of which was therapy-related (12,13). However, infection- and therapy-associated HLH were thoroughly excluded in our patient.

HLH is a poor prognostic factor for patients with hematological cancer (14–16). It is possible that the development of leukemia in patients with genetic HLH mutations may trigger overt HLH, particularly when combined with infections. The patient in this case presented with chromosomal abnormalities, which may have induced the HLH and AML. Patients with abnormalities in FHLH genes may have defective immune surveillance of abnormal clones and, as a result, may be predisposed to leukemia (17), as is likely in the present case. There is no consensus on the treatment of HLH when it is concomitant with AML. Jordan *et al* recommend firstly initiating immunochemotherapy aimed at controlling the inflammation, and then administering disease-specific therapy once inflammatory markers have normalized (13). However, in the patient in the present case, the AML was treated first, which in turn resulted in resolution of the hemophagocytosis.

In conclusion, single chemotherapy for AML with HLH was proven to be effective in the present case. The present case report has demonstrated successful chemotherapeutic treatment of a patient with AML-associated HLH. However, there are few reported cases of AML occurring with HLH in the literature and, therefore, this finding requires further investigation in a similar setting.
